# Changes of Arterial Pulse Waveform Characteristics with Gestational Age during Normal Pregnancy

**DOI:** 10.1038/s41598-018-33890-1

**Published:** 2018-10-22

**Authors:** Kunyan Li, Song Zhang, Lin Yang, Hongqing Jiang, Zhenyu Chi, Anran Wang, Yimin Yang, Xuwen Li, Dongmei Hao, Lei Zhang, Dingchang Zheng

**Affiliations:** 1 College of Life Science and Bioengineering, Beijing University of Technology, Beijing, 100124 China; 2 Haidian Maternal & Child Health Hospital, Beijing, 100026 China; 3 Department of Medical Science and Public Health, Faculty of Medical Science, Anglia Ruskin University, Chelmsford, CM1 1SQ UK

## Abstract

Arterial pulse waveform analysis has been widely used to reflect physiological changes in the cardiovascular system. This study aimed to comprehensively investigate the changes of waveform characteristics of both photoplethysmographic (PPG) and radial pulses with gestational age during normal pregnancy. PPG and radial pulses were simultaneously recorded from 130 healthy pregnant women at seven gestational time points. After normalizing the arterial pulse waveforms, the abscissa of notch point, the total pulse area and the reflection index were extracted and compared between different measurement points and between the PPG and radial pulses using post-hoc multiple comparisons with Bonferrioni correction. The results showed that the effect of gestational age on all the three waveform characteristics was significant (all p < 0.001) after adjusting for maternal age, heart rate and blood pressures. All the three waveform characteristics demonstrated similar changing trends with gestational age, and they were all significantly different between the measurements from gestational week 12–15 and the others (all p < 0.05, except for the PPG total pulse area between the first and second measurement points). In conclusion, this study has comprehensively quantified similar changes of both PPG and radial pulse waveform characteristics with gestational age.

## Introduction

Pregnant women undergo remarkable physiological changes, which occur after conception and affect many organ systems in the body, including the cardiovascular system^1–3^. These changes are essential to support the fetus development and also to prepare the mother for parturition. It is therefore clinically and physiologically important to understand how they change during normal pregnancy.

Peripheral arterial pulses have been widely used to reflect physiological changes of cardiovascular system^4–6^. The analysis of peripheral arterial pulse wave propagation and reflection can provide valuable information about the circulatory changes associated with hypertension and cardiovascular diseases. The most frequently measured peripheral arterial pulses are photoplethysmographic (PPG) and radial pulses. PPG pulse has been widely used to measure the blood oxygen saturation and used as a promising technique for predicting various cardiovascular disease^7,8^, and radial arterial pulse has been commonly used to assess the cardiac rhythm and other physiological changes.

During pregnancy, maternal physiological changes, including both the structural vascular damage and reversible alterations in vascular compliance, have been detected from arterial pulse measurement^9,10^. Pulse wave velocity (PWV) or augmentation index of aortic pulse has been commonly measured in the published studies^11–13^. Torrado *et al*. assessed and compared reactive hyperemia-related difference in carotid-radial PWV between pregnant women and non-pregnant women, and concluded that carotid-radial PWV had a potential role in assessing endothelial function during pregnancy^14^. Khalil *et al*. reported the normal values of PWV and augmentation index reflecting maternal hemodynamic in normal pregnancies at gestational week 11–13^15^. Su *et al*. longitudinally investigated the maternal cardiovascular alterations using PPG pulse and compared their differences between three trimesters during normal gestation^16^. However, the majority of those published studies mainly focused on the PWV changes or the central arterial pulse waveform changes during pregnancy, not on the peripheral pulse waveform characteristics. This is one of the aims of this study. The typical waveform characteristics derived from peripheral pulses include the location of the notch point, the total pulse area and reflection index (RI). Additionally, it is accepted that the underlying principles of optical PPG and arterial pressure pulse could be different^17–19^. To the best of our knowledge, there is no study to compare the difference of arterial pulse waveform changes during normal pregnancy between simultaneously measured PPG and radial pulses.

Moreover, the published studies investigating the maternal physiological changes were mainly conducted to compare the difference between pregnant women and non-pregnant women at one or a few gestational weeks. Khalil *et al*. performed the experiment only at gestational week 11–13^15^. Mika *et al*. reported central aortic blood pressure (BP) and augmentation index of central arteries from pregnant women at four measurement points, including gestational week 12–14, week 17–20, week 23–27 and week 34–36^20^. It would be useful to have a comprehensive longitudinal study with more measurement points to understand the maternal changes of arterial pulse waveform over the whole pregnancy.

The aim of this study was to comprehensively investigate the changes of waveform characteristics derived from both PPG and radial pulses measured at seven gestational time points (week 12–15, week 16–19, week 20–23, week 24–27, week 28–31, week 32–35, week 36–40) during normal pregnancy.

## Results

### Changes of heart rate and blood pressure during pregnancy

**Figure 1:**
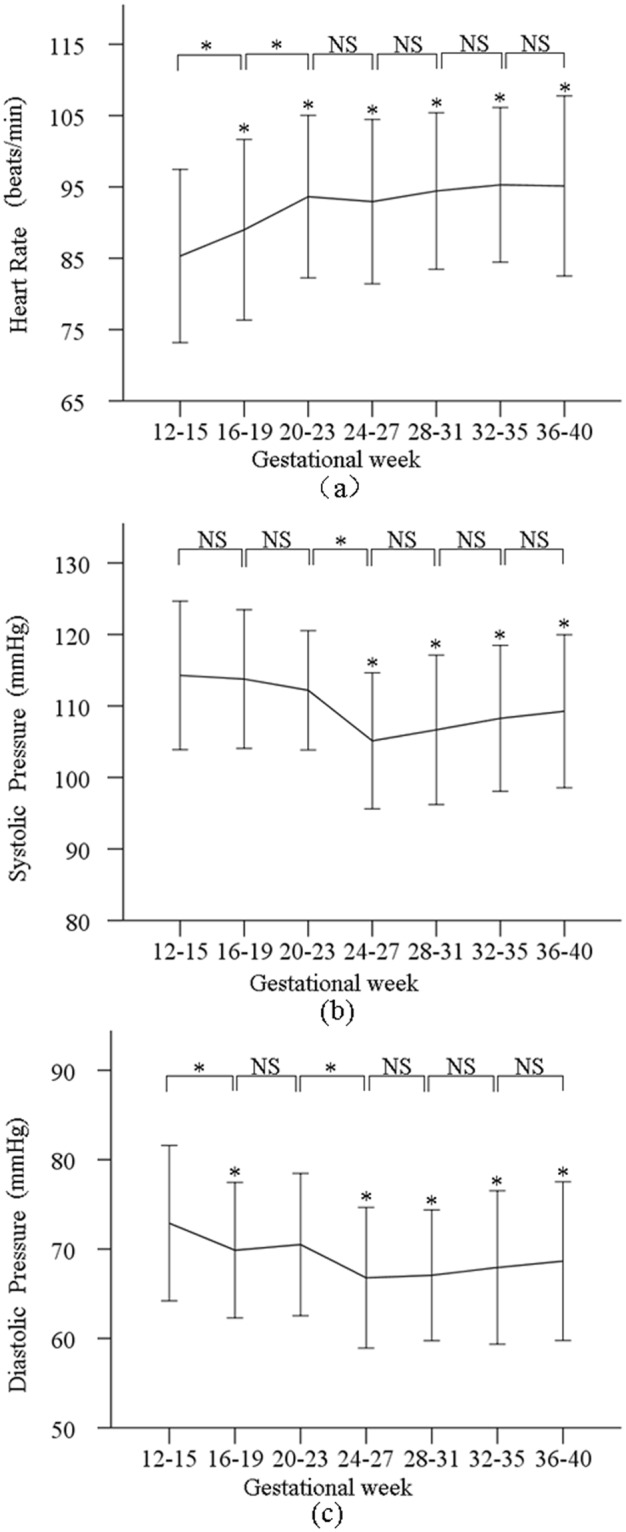
Means and SDs of the heart rate (**a**) systolic (**b**) and diastolic blood pressures (**c**) measured at the seven gestational time points during normal pregnancy.

As shown in Fig. 1, the overall heart rate (HR) had an increasing trend with gestational age. There was significant difference in both HR and systolic and diastolic blood pressures (SBP and DBP) between the measurements from gestational week 12–15 and the others (all p < 0.05, except for the SBP between the first and second, third measurement points, and the DBP between the first and third measurement points). HR increased from week 12–15 then reached a relatively stable level at week 20–23 until week 36–40. In terms of BP changes during pregnancy, SBP decreased firstly from week 12–15 to week 24–27 (114.3 ± 10.4 vs 105.0 ± 9.5 mmHg, p < 0.001) where the lowest SBP was observed, and then increased slowly back to 109.0 ± 10.7 mmHg at week 36–40 (p < 0.001). For DBP, a similar changing trend was observed with the lowest DBP observed at gestational week 24–27 (66.8 ± 7.9 mmHg).

### Change of overall normalized pulse shape during pregnancy

**Figure 2:**
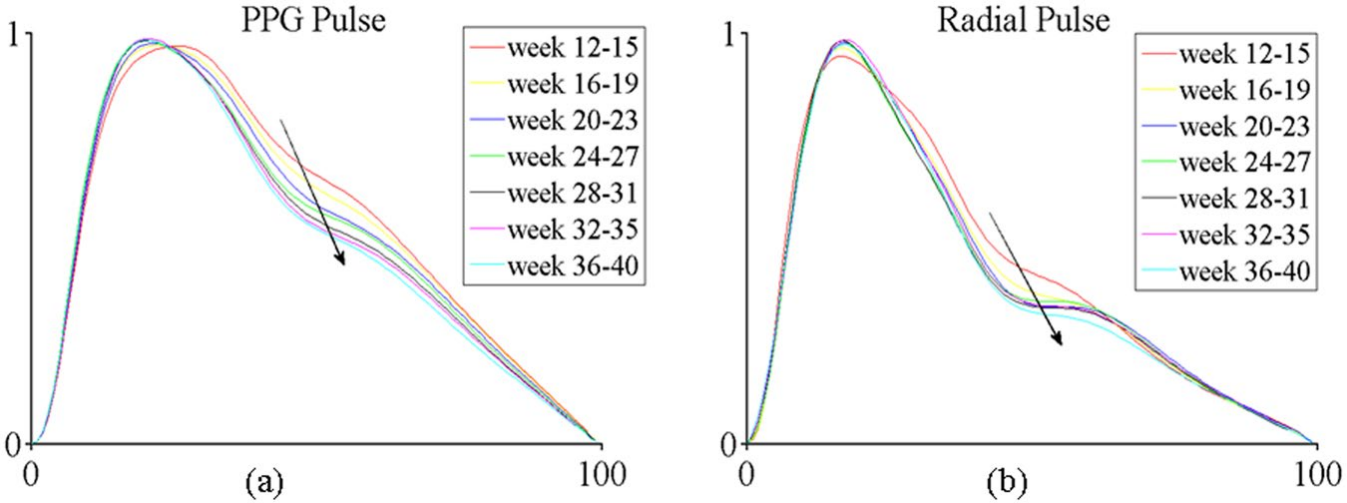
Illustration of normalized PPG (**a**) and radial (**b**) pulse waveforms at the seven gestational time points during normal pregnancy. The averaged waveform across all the pregnant women is plotted.

Figure 2 illustrates the overall normalized PPG and radial pulse waveforms at the seven gestational time points during normal pregnancy. It can be seen that the abscissa of the notch point (Tn) of both PPG and radial pulses moved to the right and downward during pregnancy, indicating that the Tn would increase and RI would decrease with increasing gestational week.

### Change of total pulse area during pregnancy

**Figure 3:**
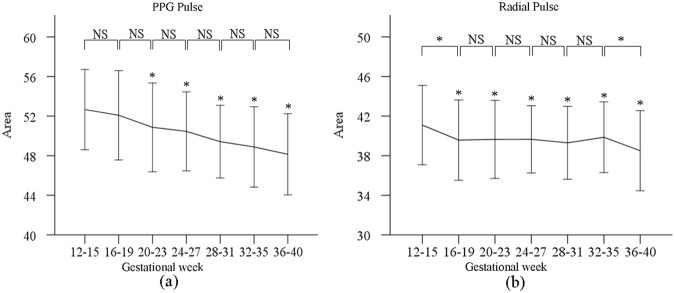
Means and SDs of the total pulse area from PPG (**a**) and radial pulses (**b**) at the seven gestational time points during normal pregnancy.

Figure 3 shows the total area of normalized PPG and radial pulses at the seven gestational time points. The post-hoc multiple comparisons with Bonferroni correction revealed that the effect of gestational age on the total pulse area (for both PPG and radial pulses) was significant (both p < 0.001) after adjusting for maternal age, HR and BPs. The total pulse area of PPG and radial pulses decreased from gestational week 12–15 to gestational week 36–40, with significant differences between week 12–15 and the other gestational time points (all p < 0.05, except for the week 16–19 of PPG pulse). In detail, the maximum adjusted total PPG pulse area was 52.6 ± 4.1 at week 12–15, and the minimum adjusted value was 48.2 ± 4.1 at week 36–40. For the radial pulses, their corresponding values were 41.0 ± 4.0 and 38.5 ± 4.1 at gestational week 12–15 and week 36–40, respectively.

### Change of Tn during pregnancy

**Figure 4:**
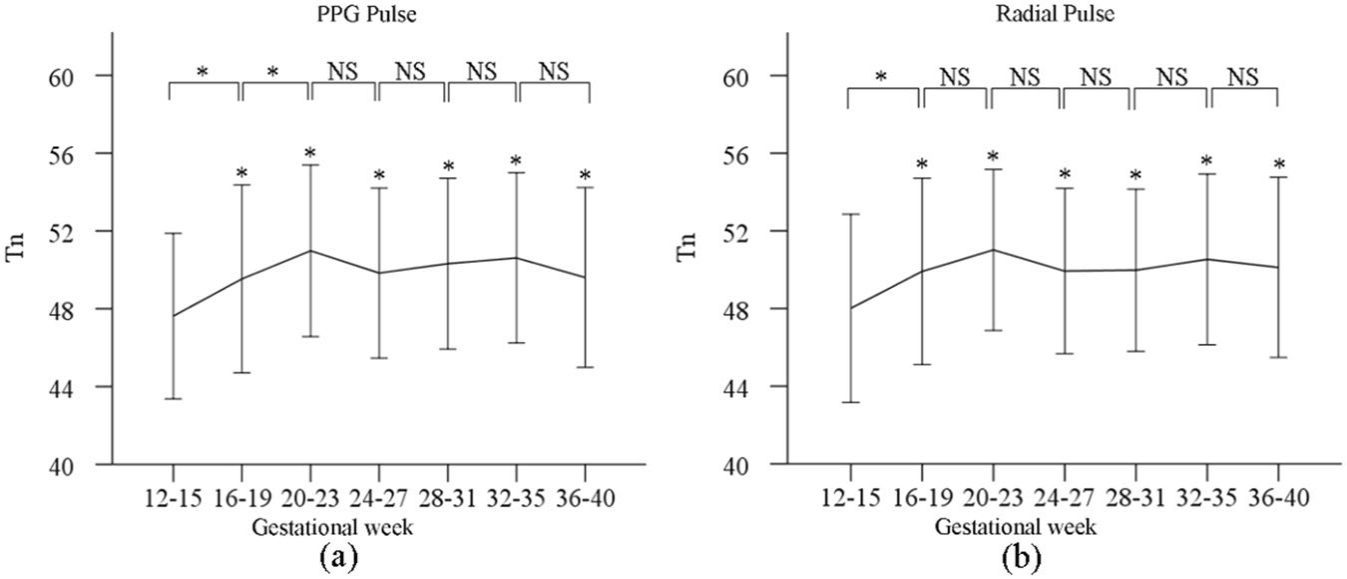
Means and SDs of the Tn from normalized PPG (**a**) and radial pulses (**b**) at the seven gestational time points during normal pregnancy.

Figure 4 shows the derived Tn values from both normalized PPG and radial pulses at the seven gestational time points. The post-hoc multiple comparisons with Bonferroni correction revealed that the effect of gestational age on Tn (for both PPG and radial pulses) was significant (both p < 0.001) after adjusting for maternal age, HR and BPs. As shown in Fig. 4, adjusted Tn of both PPG and radial pulses increased from gestational week 12–15 (PPG: 47.5 ± 4.3 at week 12–15 vs 49.6 ± 4.6 at week 36–40, p < 0.001; corresponding values from radial pulses: 47.9 ± 4.8 vs 50.1 ± 4.6, p < 0.001). There were significant differences between gestational week 12–15 and the other gestational time points (all p < 0.001). After comparing the Tn changes with gestational week between PPG and radial pulses, it was observed that there were similar changing pattern between any two consecutive gestational time points, except for the comparison between gestational week 16–19 and week 20–23 (p = 0.03 for PPG pulse, p = 0.3 for radial pulse).

### Changes of RI during pregnancy

**Figure 5:**
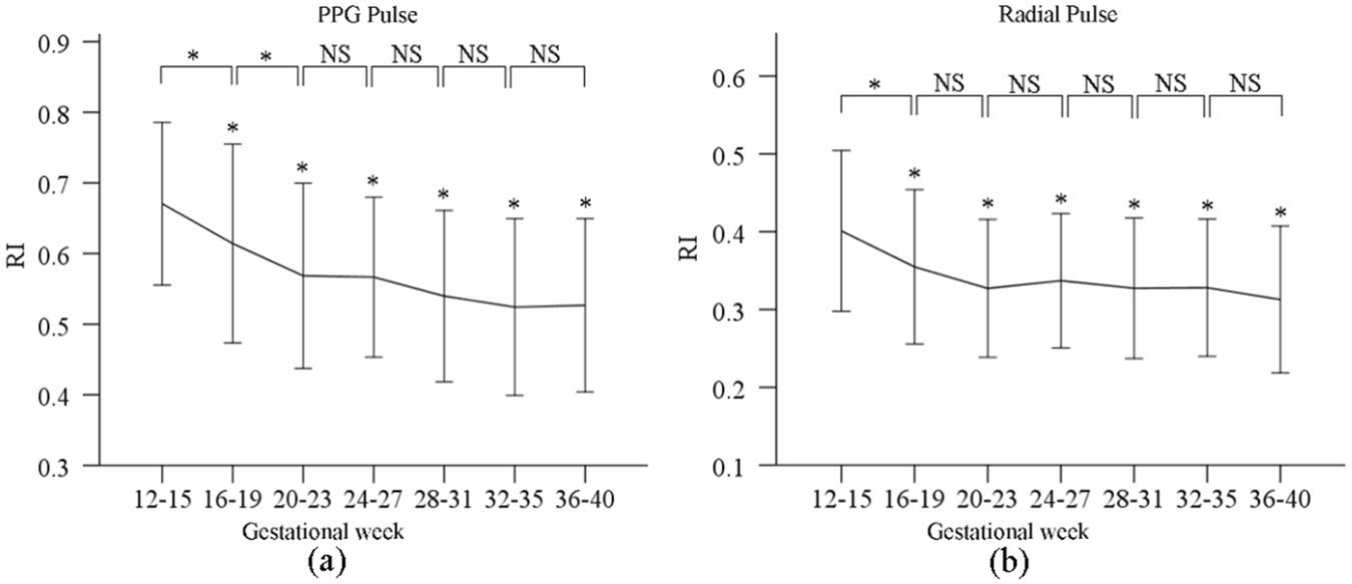
Means and SDs of the RI derived from normalized PPG (**a**) and radial pulses (**b**) at the seven gestational time points during normal pregnancy.

Figure 5 shows the RI from both normalized PPG and radial pulses at the seven gestational time points. The post-hoc multiple comparisons with Bonferroni correction revealed that the effect of gestational age on RI (for both PPG and radial pulses) was significant (both p < 0.001) after adjusting for maternal age, HR and BPs. In detail, there were significant RI differences between week 12–15 and the other gestational time points (all p < 0.001). Adjusted RI decreased from gestational week 12–15 to week 36–40, with the maximum value from PPG of 0.7 ± 0.1 at week 12–15 and the minimum value from PPG of 0.5 ± 0.1 at week 36–40. Their corresponding maximum and minimum RIs from the radial pulses were 0.4 ± 0.1 at gestational week 12–15 and 0.3 ± 0.1 at gestational week 36–40. Again, similar changing trends between any two consecutive gestational time points were observed between the PPG and radial pulses, except for the comparison between gestational week 16–19 and week 20–23 (p = 0.01 for PPG pulse, p = 0.09 for radial pulse).

## Discussion and Conclusion

In this study, three arterial pulse waveform characteristics (the total pulse area, Tn, RI) derived from normalized PPG and radial pulses have been quantified and compared between seven longitudinal gestational time points during normal pregnancy. To the best of our knowledge, this is the first study to comprehensively investigate both the peripheral PPG and radial pulse waveform changes with seven gestational time points during normal pregnancy.

The first observation in this study was that the maternal HR increased, and BPs decreased during the early stage of pregnancy from week 12–15 and then reached their corresponding maximum and minimum values at week 24–27. This agreed with the published study by Andrew *et al*., where decreased BPs were reported in early period of pregnancy as a compensatory response to falling systemic vascular resistance^21^. An increase in maternal HR is possibly associated with increased cardiac output and stroke volume^22^.

Secondly, decreased total pulse area has been observed from both the PPG and radial pulse measurements during normal pregnancy. Wang *et al*. reported that the changes of radial total pulse area were associated with the physiological changes^23^. Nirmalan *et al*. and Greenwald *et al*. reported that a narrowed main wave and dicrotic wave were caused by increased cardiac ejection in association with decreased peripheral vascular resistance, resulting in lower diacritic notch point^24,25^. According to this principle, the physiological changes in ejection function, peripheral resistance and blood vessel elasticity during pregnancy has been reflected in the total pulse area changes as demonstrated in this study^25^.

This study also demonstrated that the Tn of both the PPG and radial pulses increased during pregnancy. It has been reporeted that the Tn of peripheral pulses is the result of the superposition of incident and reflecting waves^26,27^. Published studies also showed the position of notch point possibly change with the blood viscosity during pregnancy^28–30^. It is therefore worthy of further investigation to better understand the underlying physiological mechanism behind the waveform characteristics changes of pregnant women.

Moreover, it was observed that the RI of both PPG pulse and radial pulses decreased gradually during normal pregnancy. RI from peripheral pulses can be used to measure peripheral arterial stiffness to provide information on mechanical properties and endothelial function of arteries under different clinical scenarios like diabetic, hypertensive patients, and postmenopausal women^31–33^. The larger the RI is, the stiffer the peripheral artery is^34^. Decreased RI with gestational week indicates the peripheral arteries become more compliant during pregnancy. Our results partially agreed with some published studies where the changes of arterial properties during pregnancy were studied using other indices. Mika *et al*. and Khalil *et al*. reported that central aortic HR-corrected augmentation index (AIx-75) decreased firstly and reached a nadir at middle phase of pregnancy, then increased at the late stage of pregnancy^20,35^, indicating that the aortic elasticity become better and then worse during normal pregnancy. Similar changing pattern in both carotid PWV and brachial-ankle PWV was also observed during normal pregnancy by Yuan *et al*. and Mizuho *et al*.^36,37^. However, the majority of these published studies mainly focused on the large arteries, rather than the middle-sized peripheral arteries, and the changes of arterial properties with physiological changes could be different depending on the measurement site. It is therefore worth investigating the arterial pulse waveform characteristics difference with measurements from different arteries during pregnancy in future studies. Agnoletti *et al*. reported that the characteristics derived from aortic waveforms are also useful and powerful in assessing cardiovascular function^38,39^. Keerthana *et al*. also reported that non-invasive central waveforms could be estimated from peripheral arterial measurements by using transfer function^40^. It would be useful to further study the central pulse waveform characteristics estimated from peripheral artery during normal pregnancy.

Finally, it has been reported that both the radial and PPG pulses have been frequently used to assess cardiovascular function^41,42^, and their waveform changes were similar in reflecting cardiovascular function changes. It is accepted that the underlying mechanisms of the optical PPG and arterial pressure pulses could be different^17–19^. In this study, similar changing trends with gestational age were also observed in all the three waveform parameters derived from both normalized radial and normalized PPG pulses, suggesting that both pulse measurement techniques are similarly useful in monitoring maternal physiological changes.

In summary, this study has comprehensively quantified the changes of PPG and radial pulse waveform characteristics throughout the normal pregnancy from gestational week 12, providing valuable information to better understand the cardiovascular physiological changes during normal pregnancy.

## Methods

### Subject information

130 healthy pregnant women volunteers, with average age of 30.8 ± 3.6 years, height of 162 ± 6 cm, weight of 56.3 ± 8.3 kg and body mass index of 21.4 ± 2.6 kg/m^2^, were recruited at the Haidian Maternal & Child Health Hospital, Beijing. All pregnant women were followed up from week 12 of pregnancy. Seven visits to the hospital for arterial pulse measurements were required at week 12–15; week 16–19; week 20–23; week 24–27; week 28–31; week 32–35; week 36–40.

This study was approved by the Ethics Committee of Beijing University of Technology. All experiments were performed in accordance with the Declaration of Helsinki. After reading the Participant Consent Form, each individual volunteer understood the purpose of this study and agreed to take part in, then gave written informed consent before inclusion in the study. Before the experiment, volunteers were abstained from alcohol, caffeine and any drugs. Pregnant women with multiple pregnancies, abnormal menstrual cycle, chronic hypertension, diabetes, anemia and any other known diseases during pregnancy were excluded from this study.

### Arterial pulse measurement procedure

The arterial pulse measurements were performed in a quiet clinical measurement room at the Haidian Maternal & Child Health Hospital, Beijing, China. During each of the seven visits, all the pregnant women were asked to sit quietly for 5 minutes to achieve stable cardiovascular status before formal recording of arterial pulse. Resting BPs and HR were firstly measured from each individual using a validated electronic sphygmomanometer (HEM-7124 from Omron Crop.). With the pregnant woman in a supine position, PPG was detected by a photoelectric sensor on the left index finger, and radial pulse was detected by a pressure sensor on the left wrist. They were recorded simultaneously by the PowerLab data collection system (ADInstruments Pty Ltd., PowerLab 8/35, Bella Vista NSW 2153, Australia) with a sampling frequency of 1000 Hz and 16-bit A/D. In total, there were seven PPG and seven radial pulse recordings from each of the 130 pregnant women.

### Determination of arterial pulse wave characteristics

**Figure 6:**
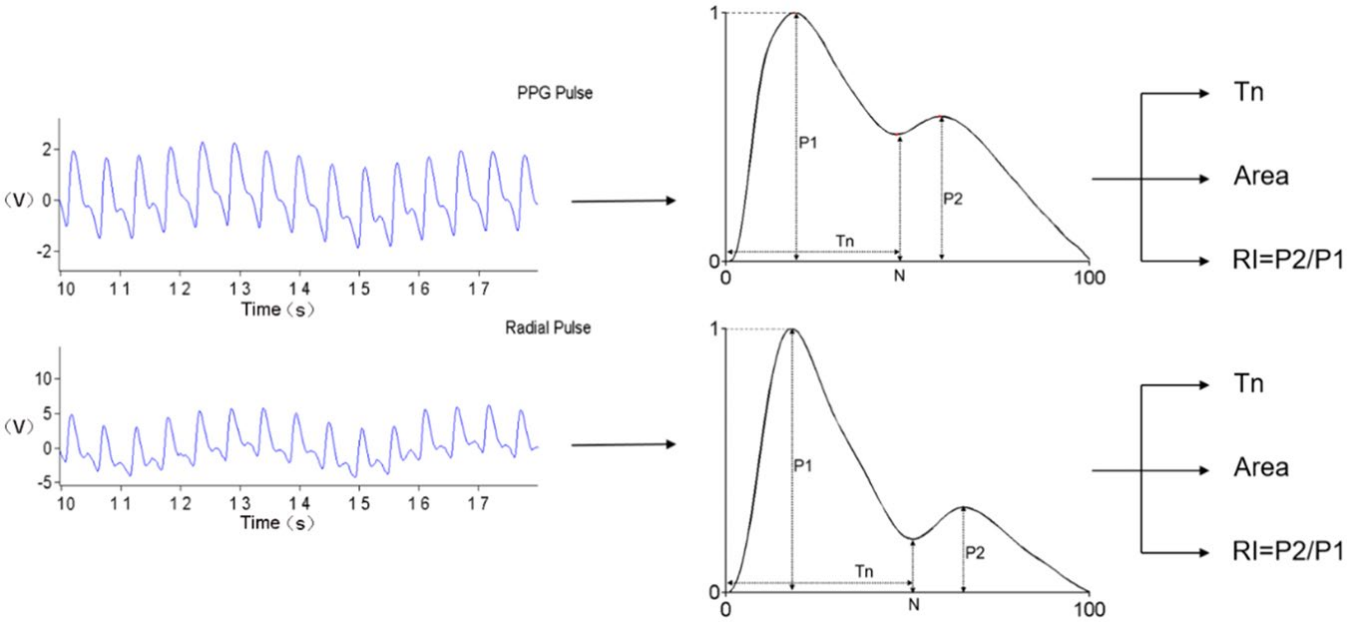
Definition of arterial pulse waveform characteristics derived from normalized radial and PPG pulses.

All the recorded radial and PPG pulses from each measurement were firstly processed to remove baseline drift. They were then processed to segment several single pulse waveforms corresponding to each heartbeat. These single pulse waveforms were normalized in both width (100 sampling points) and amplitude (0–1) from the foot of each pulse and then averaged to obtain a single normalized pulse for each measurement, as shown in Fig. 6.

Three waveform characteristics were extracted from the normalized single waveform, including the ‘total Pulse Area’ under the waveform, the Tn (the abscissa of the pulse notch point), and the RI (RI = P2/P1), as proposed by Wang *et al*.^34^. These processes were done using MATLAB (version R2014a).

The total pulse area that describes the global pulse wave characteristics was computed from the normalized pulse waveform as: ${\rm{Total}}\,{\rm{area}}={\int }_{0}^{100}Y(t)dt$. Luo *et al*. and Li *et al*. reported that it reflected the variation of peripheral resistance, vascular wall elasticity and viscosity^43,44^.

Tn is associated with the reflection of peripheral pulse waveform, reflecting the elasticity of the small arteries. RI is a measure of arterial stiffness, which can be influenced by wave reflections from the peripheral arteries, and reflects the alterations in muscular smooth muscle tone of peripheral arteries. RI may be an indirect measure of vasoconstriction^45^.

### Data statistical analysis

The means ± SDs of all the basic clinical parameters (HR and BPs) and the derived arterial pulse waveform characteristics (PPG pulse Tn, PPG total pulse area, PPG pulse RI, radial pulse Tn, radial total pulse area, and radial pulse RI) were calculated across all the pregnant women, separately for the seven gestational time points. Analysis of covariance was then performed using SPSS to investigate the effect of gestational week on all the PPG and radial pulse waveform characteristics after adjusting for maternal age, HR, and BPs. The post-hoc multiple comparisons with Bonferroni correction was further applied to determine significant difference between pairs (p for any pair had to be smaller than 0.05/n, n was all the possible pairs. n = 21). A p < 0.05 was used as the significant criterion.

## Data Availability

All data generated or analyzed during the current study are available from the corresponding author on reasonable request.
